# 5-{[4-(Di­methyl­amino)­phen­yl]ethyn­yl}pyrimidine–1,2,3,5-tetra­fluoro-4,6-di­iodo­benzene (1/2)

**DOI:** 10.1107/S2414314622003807

**Published:** 2022-04-12

**Authors:** Eric Bosch, Nathan P. Bowling

**Affiliations:** a Chemistry and Biochemistry Department, Missouri State University, Springfield MO 65897, USA; b Department of Chemistry, University of Wisconsin-Stevens Point, 2101 Fourth Avenue, Stevens Point, WI 54481, USA; University of Aberdeen, Scotland

**Keywords:** crystal structure, halogen bonding, co-crystal, 1,2,3,5-tetra­fluoro-4,6-di­iodo­benzene solvate

## Abstract

The treatment of 5-{[4-(di­methyl­amino)­phen­yl]ethyn­yl}pyrimidine with a threefold excess of 1,2,3,5-tetra­fluoro-4,6-di­iodo­benzene in di­chloro­methane solution led to the formation of the unexpected 1:2 title co-crystal, C_14_H_13_N_3_·2CF_4_I_2_. In the extended structure, two unique C—I⋯N halogen bonds from one of the 1,2,3,5-tetra­fluoro-4,6-di­iodo­benzene mol­ecules to the pyrimidine N atoms of the 5-{[4-(di­methyl­amino)­phen­yl]ethyn­yl}pyrimidine mol­ecule generate [110] chains and layers of these chains are π-stacked along the *a-*axis direction. The second 1,2,3,5-tetra­fluoro-4,6-di­iodo­benzene mol­ecule resides in channels formed parallel to the *a*-axis direction between stacks of 5-{[4-(di­methyl­amino)­phen­yl]ethyn­yl}pyrimidine mol­ecules and inter­acts with them *via* C—I⋯π(alkyne) contacts.

## Abstract 2

In the title 1:2 co-crystal, C—I⋯N halogen bonds between one of the 1,2,3,5-tetra­fluoro-4,6-di­iodo­benzene mol­ecules and the 5-{[4-(di­methyl­amino)­phen­yl]ethyn­yl}pyrimidine mol­ecule form [110] chains while the second 1,2,3,5-tetra­fluoro-4,6-di­iodo­benzene mol­ecule resides in [100] channels.

## Graphical abstract

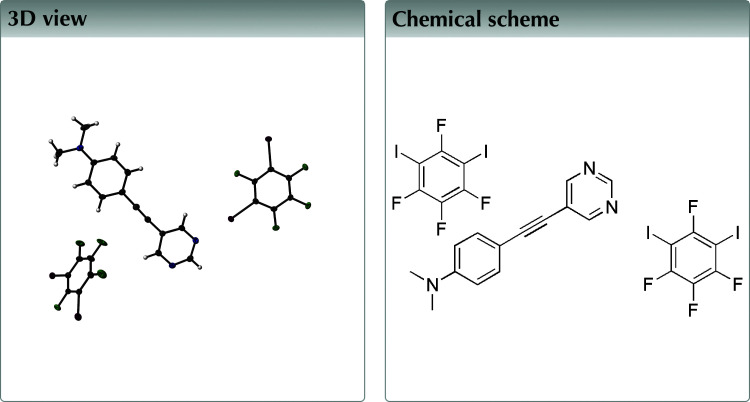

## Structure description

Halogen bonding is now a widely studied and accepted non-covalent inter­action wherein a halogen atom, most commonly iodine, inter­acts with a Lewis base as halogen-bond acceptor (Cavallo *et al.*, 2016 ▸). This inter­action has predictable geometry and has accordingly been incorporated in strategies for the self-assembly of multicomponent mol­ecular solids (Mir *et al.*, 2019 ▸). Among the most studied ditopic halogen-bond donors are the three isomeric di­iodo­tetra­fluoro­benzenes as the halogen-bond donor ability is increased by substitution of iodo­benzenes with electronegative fluorine atoms (Roper *et al.*, 2010 ▸). Herein we report a rare example of inclusion of a 1,2,3,5-tetra­fluoro-4,6-di­iodo­benzene mol­ecule in a co-crystal in which one of the 1,2,3,5-tetra­fluoro-4,6-di­iodo­benzene mol­ecules does not inter­act with the primary Lewis base.

**Figure 1:**
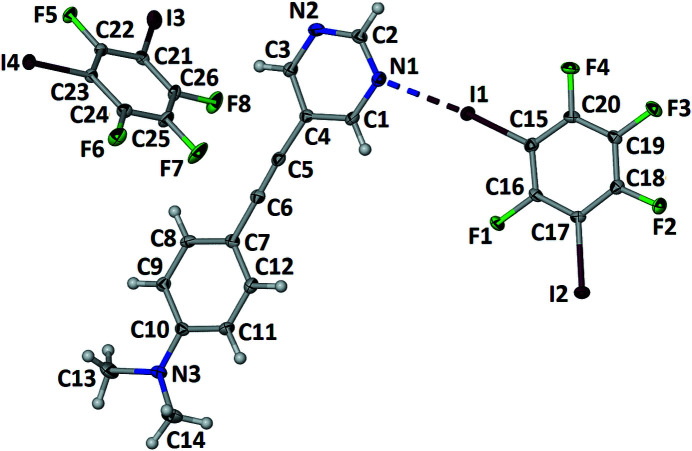
The mol­ecular structure of the title compound with displacement ellipsoids drawn at 50% and the halogen bond shown as a dashed line.

**Figure 2:**
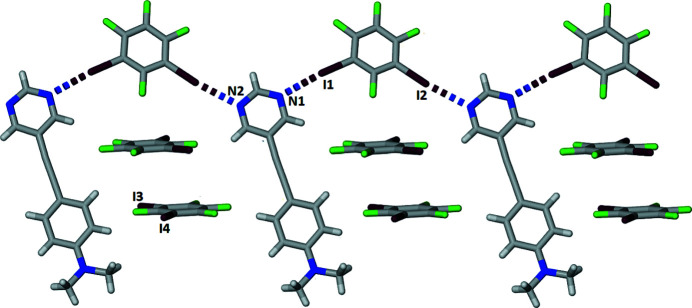
Partial view of a chain of halogen-bonded APEP and 13DIFB mol­ecules with pairs of 13DIFB mol­ecules shown between APEP mol­ecules.

**Figure 3:**
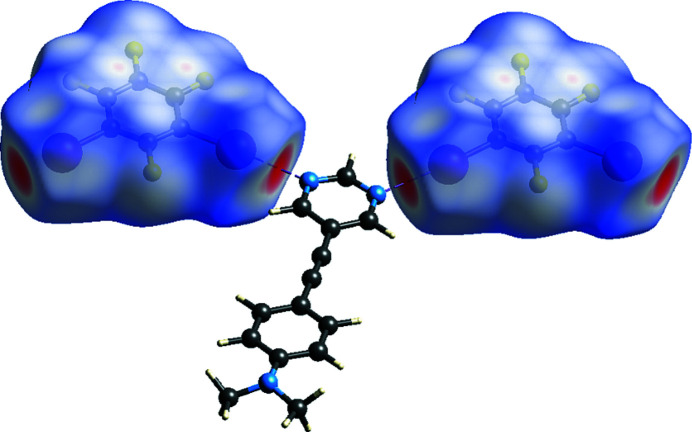
Hirshfeld surface highlighting the halogen-bonding inter­actions to pyrimidine APEP.

In the 1:2 co-crystal (Fig. 1 ▸) formed between 5-{[4-(di­methyl­amino)­phen­yl]ethyn­yl}pyrimidine, C_14_H_13_N_3_ (APEP) and 1,2,3,5-tetra­fluoro-4,6-di­iodo­benzene, C_6_F_4_I_2_ (13DIFP), only one of the 13DIFP mol­ecules is halogen bonded to the APEP. The APEP and the halogen-bonded 13DIFP mol­ecule are essentially coplanar: the inter­planar angle between the pyrimidine ring and the amino­phenyl ring is 4.24 (15)° and the inter­planar angle between the pyrimidine ring and the halogen-bonded 13DIFP mol­ecule is 6.63 (15)°. The two unique C—I⋯N halogen bonds that combine to form a zigzag alternating halogen-bonded chain, shown in Fig. 2 ▸, have separations of I1⋯N1 and I2⋯N2^i^ = 2.853 (2) and 2.901 (2) Å and angles C15—I1⋯N1 and C17—I2⋯N2^i^ = 174.8 (9) and 173.8 (8)°, respectively [symmetry code: (i) −1 + *x*, −1 + *y*, *z*]. These distances and angles are similar to those previously reported in the 1:1 co-crystal formed between these two mol­ecules of 2.920 (2) Å and 178.27 (6)° (Nwachukwu *et al.*, 2020 ▸). The Hirshfeld surface (Spackman *et al.*, 2021 ▸) of the halogen-bonded 13DIFB mol­ecules shown in Fig. 3 ▸ highlights these two inter­actions.

**Figure 4:**
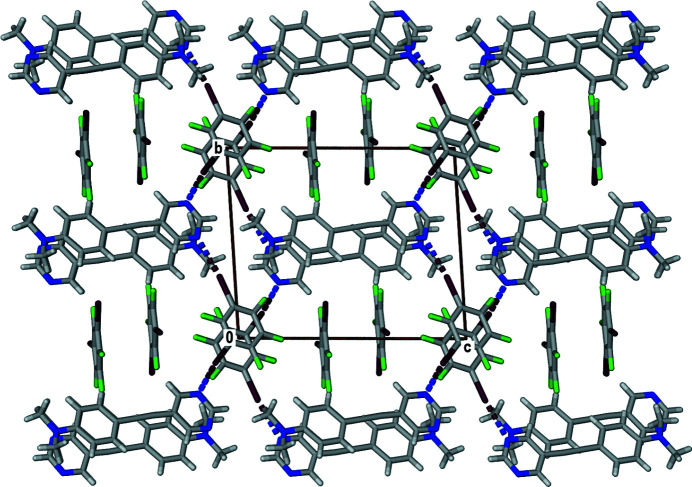
View of crystal packing of the title compound viewed along the *a-*axis direction.

In the extended structure, the APEP mol­ecules are offset π-stacked in a head-to-tail manner such that the halogen-bonded 13DIFB mol­ecules are also alternately π-stacked as shown in Fig. 4 ▸. With this arrangement, the second non-halogen-bonded 13DIFB mol­ecule is located as a π-stacked pair in channels that lie parallel to the *a-*axis direction (Fig. 4 ▸).

**Figure 5:**
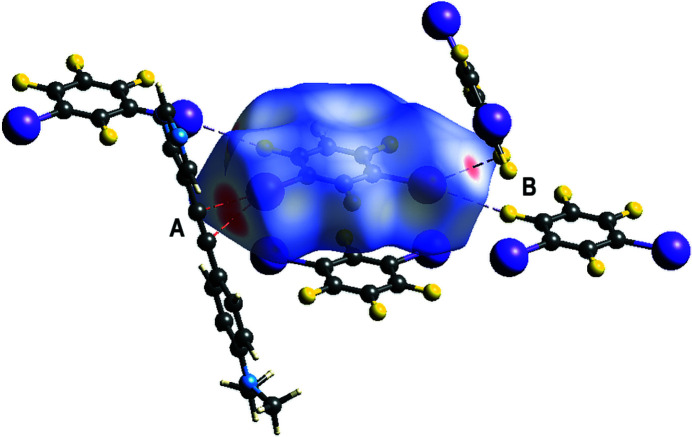
Hirshfeld surface highlighting the close contacts to the solvate 13DIFB mol­ecule.

The pair of loosely π-stacked 13DIFB mol­ecules inter­act with the surrounding mol­ecules as shown in the Hirshfeld surface plot in Fig. 5 ▸. This highlights a close I⋯π contact to a neighboring alkyne group with I4⋯C6^ii^ and I4⋯C5^ii^ [symmetry code: (ii) 1 − *x*, 2 − *y*, 1 − *z*] separations of 3.276 (3) and 3.316 (3) Å, respectively. These are significantly less than the sum of the van der Waals radii of 3.68 Å at 89 and 90%, respectively. The second I atom has close I⋯F contacts to two neighboring 13DIFB mol­ecules with I3⋯F6^iii^ and I3⋯F3^iv^ separations of 3.2142 (17) and 3.30129 (15) Å as compared to the sum of the van der Waals radii of 3.38 Å [symmetry codes: (iii) 1 + *x*, *y*, *z*; (iv) *x*, 1 + *y*, −1 + *z*].

## Synthesis and crystallization

The pyrimidine APEP (8.3 mg) was dissolved in 2 ml of di­chloro­methane in a screw-cap vial. Three equivalents of 13DIFB were added and the solvent was allowed to slowly evaporate until crystals formed when the vial was sealed to prevent further loss of solvent.

## Refinement

Crystal data, data collection and structure refinement details are summarized in Table 1 ▸.

## Supplementary Material

Crystal structure: contains datablock(s) I. DOI: 10.1107/S2414314622003807/hb4404sup1.cif

Structure factors: contains datablock(s) I. DOI: 10.1107/S2414314622003807/hb4404Isup2.hkl

Supporting information file. DOI: 10.1107/S2414314622003807/hb4404Isup3.cdx

Supporting information file. DOI: 10.1107/S2414314622003807/hb4404Isup4.cml

CCDC reference: 2164881

Additional supporting information:  crystallographic information; 3D view; checkCIF report

## Figures and Tables

**Table 1:** Experimental details

Crystal data	
Chemical formula	C_14_H_13_N_3_·2C_6_F_4_I_2_
*M*_r_	1026.99
Crystal system, space group	Triclinic, *P* 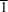
Temperature (K)	100
*a*, *b*, *c* (Å)	9.1574 (5), 12.0339 (6), 14.0667 (7)
α, β, γ (°)	91.989 (1), 96.924 (1), 102.996 (1)
*V* (Å^3^)	1496.35 (13)
*Z*	2
Radiation type	Mo *K*α
μ (mm^−1^)	4.24
Crystal size (mm)	0.52 × 0.28 × 0.20

Data collection	
Diffractometer	Bruker APEXI CCD
Absorption correction	Multi-scan (*SADABS*; Bruker, 2014 ▸)
*T*_min_, *T*_max_	0.518, 0.746
No. of measured, independent and observed [*I* > 2σ(*I*)] reflections	19410, 6594, 6107
*R*_int_	0.023
(sin θ/λ)_max_ (Å^−1^)	0.641

Refinement	
*R*[*F* ^2^ > 2σ(*F* ^2^)], *wR*(*F* ^2^), *S*	0.020, 0.046, 1.09
No. of reflections	6594
No. of parameters	372
H-atom treatment	H-atom parameters constrained
Δρ_max_, Δρ_min_ (e Å^−3^)	0.69, −0.56

Computer programs: *SMART* and *SAINT* (Bruker, 2014 ▸), *SHELXT2018/2* (Sheldrick, 2015*a* ▸), *SHELXL2018/3* (Sheldrick, 2015*b* ▸), and *X-SEED* (Barbour, 2020 ▸).

## full crystallographic data

### Crystal data

**Table:**

C_14_H_13_N_3_·2C_6_F_4_I_2_	*Z* = 2
*M**_r_* = 1026.99	*F*(000) = 948
Triclinic, *P*1	*D*_x_ = 2.279 Mg m^−^^3^
*a* = 9.1574 (5) Å	Mo *K*α radiation, λ = 0.71073 Å
*b* = 12.0339 (6) Å	Cell parameters from 9920 reflections
*c* = 14.0667 (7) Å	θ = 2.3–27.1°
α = 91.989 (1)°	µ = 4.24 mm^−^^1^
β = 96.924 (1)°	*T* = 100 K
γ = 102.996 (1)°	Irregular cut block, colourless
*V* = 1496.35 (13) Å^3^	0.52 × 0.28 × 0.20 mm

### Data collection

**Table:**

Bruker APEXI CCD diffractometer	6594 independent reflections
Radiation source: fine-focus sealed tube	6107 reflections with *I* > 2σ(*I*)
Graphite monochromator	*R*_int_ = 0.023
Detector resolution: 8.3660 pixels mm^-1^	θ_max_ = 27.1°, θ_min_ = 2.2°
phi and ω scans	*h* = −11→11
Absorption correction: multi-scan (SADABS; Bruker, 2014)	*k* = −15→15
*T*_min_ = 0.518, *T*_max_ = 0.746	*l* = −17→18
19410 measured reflections	

### Refinement

**Table:**

Refinement on *F*^2^	Primary atom site location: dual
Least-squares matrix: full	Hydrogen site location: inferred from neighbouring sites
*R*[*F*^2^ > 2σ(*F*^2^)] = 0.020	H-atom parameters constrained
*wR*(*F*^2^) = 0.046	*w* = 1/[σ^2^(*F*_o_^2^) + (0.0204*P*)^2^ + 0.5553*P*] where *P* = (*F*_o_^2^ + 2*F*_c_^2^)/3
*S* = 1.09	(Δ/σ)_max_ = 0.002
6594 reflections	Δρ_max_ = 0.69 e Å^−^^3^
372 parameters	Δρ_min_ = −0.56 e Å^−^^3^
0 restraints	

### Special details

**Table:**

Geometry. All esds (except the esd in the dihedral angle between two l.s. planes) are estimated using the full covariance matrix. The cell esds are taken into account individually in the estimation of esds in distances, angles and torsion angles; correlations between esds in cell parameters are only used when they are defined by crystal symmetry. An approximate (isotropic) treatment of cell esds is used for estimating esds involving l.s. planes.

### Fractional atomic coordinates and isotropic or equivalent isotropic displacement parameters (Å^2^)

**Table:**

	*x*	*y*	*z*	*U*_iso_*/*U*_eq_	
I1	0.49323 (2)	0.32275 (2)	0.93280 (2)	0.02074 (5)	
F1	0.21264 (18)	0.11841 (13)	0.85256 (11)	0.0253 (4)	
N1	0.6063 (3)	0.52854 (19)	0.84397 (16)	0.0228 (5)	
C1	0.5221 (3)	0.5211 (2)	0.75898 (19)	0.0203 (6)	
H1	0.442392	0.455214	0.742023	0.024*	
I2	0.02348 (2)	−0.12845 (2)	0.89212 (2)	0.01983 (5)	
F2	0.23180 (18)	−0.14839 (13)	1.08815 (11)	0.0245 (3)	
N2	0.7521 (3)	0.70747 (19)	0.80721 (16)	0.0228 (5)	
C2	0.7169 (3)	0.6221 (2)	0.8639 (2)	0.0215 (6)	
H2	0.776741	0.628608	0.924803	0.026*	
I3	1.23452 (2)	0.97142 (2)	0.34507 (2)	0.02347 (5)	
F3	0.47643 (17)	−0.00515 (14)	1.18660 (11)	0.0240 (3)	
N3	−0.0078 (3)	0.5104 (2)	0.17456 (18)	0.0288 (6)	
C3	0.6668 (3)	0.6985 (2)	0.7221 (2)	0.0229 (6)	
H3	0.689419	0.757418	0.679287	0.027*	
I4	0.70571 (2)	1.18190 (2)	0.39166 (2)	0.01903 (5)	
F4	0.58522 (17)	0.20184 (14)	1.12306 (11)	0.0256 (4)	
C4	0.5460 (3)	0.6057 (2)	0.69404 (19)	0.0176 (5)	
F5	1.03550 (16)	1.15246 (12)	0.36927 (11)	0.0219 (3)	
C5	0.4513 (3)	0.5954 (2)	0.6039 (2)	0.0203 (6)	
F6	0.56017 (17)	0.91354 (14)	0.39466 (14)	0.0306 (4)	
C6	0.3687 (3)	0.5801 (2)	0.5296 (2)	0.0199 (5)	
F7	0.67827 (19)	0.72914 (14)	0.38368 (16)	0.0402 (5)	
C7	0.2714 (3)	0.5631 (2)	0.44040 (19)	0.0192 (5)	
F8	0.97066 (19)	0.75248 (14)	0.36268 (14)	0.0338 (4)	
C8	0.2961 (3)	0.6402 (2)	0.36941 (19)	0.0200 (5)	
H8	0.378242	0.705295	0.381043	0.024*	
C9	0.2039 (3)	0.6246 (2)	0.2823 (2)	0.0203 (6)	
H9	0.223836	0.678728	0.235226	0.024*	
C10	0.0812 (3)	0.5295 (2)	0.2628 (2)	0.0202 (5)	
C11	0.0557 (3)	0.4526 (2)	0.3355 (2)	0.0236 (6)	
H11	−0.027198	0.387958	0.324617	0.028*	
C12	0.1480 (3)	0.4689 (2)	0.4219 (2)	0.0239 (6)	
H12	0.127870	0.415619	0.469581	0.029*	
C13	0.0052 (4)	0.6021 (3)	0.1095 (2)	0.0473 (10)	
H13A	−0.025722	0.666905	0.138573	0.071*	
H13B	−0.060174	0.575019	0.048978	0.071*	
H13C	0.110369	0.626297	0.097130	0.071*	
C14	−0.1542 (3)	0.4298 (3)	0.1657 (3)	0.0378 (8)	
H14A	−0.139652	0.353862	0.180913	0.057*	
H14B	−0.204986	0.426549	0.099927	0.057*	
H14C	−0.216383	0.454809	0.210428	0.057*	
C15	0.4006 (3)	0.1649 (2)	0.98576 (19)	0.0193 (5)	
C16	0.2772 (3)	0.0880 (2)	0.93589 (18)	0.0179 (5)	
C17	0.2162 (3)	−0.0179 (2)	0.96755 (18)	0.0174 (5)	
C18	0.2848 (3)	−0.0463 (2)	1.05331 (19)	0.0186 (5)	
C19	0.4083 (3)	0.0263 (2)	1.10472 (18)	0.0176 (5)	
C20	0.4647 (3)	0.1320 (2)	1.07115 (19)	0.0188 (5)	
C21	1.0096 (3)	0.9535 (2)	0.36423 (18)	0.0171 (5)	
C22	0.9461 (3)	1.0476 (2)	0.37107 (18)	0.0162 (5)	
C23	0.7959 (3)	1.0382 (2)	0.38056 (18)	0.0167 (5)	
C24	0.7064 (3)	0.9287 (2)	0.3845 (2)	0.0201 (6)	
C25	0.7661 (3)	0.8338 (2)	0.3796 (2)	0.0246 (6)	
C26	0.9165 (3)	0.8463 (2)	0.3691 (2)	0.0207 (6)	

### Atomic displacement parameters (Å^2^)

**Table:**

	*U*^11^	*U*^22^	*U*^33^	*U*^12^	*U*^13^	*U*^23^
I1	0.02265 (9)	0.01765 (9)	0.01957 (9)	−0.00020 (7)	0.00227 (7)	0.00274 (7)
F1	0.0277 (9)	0.0261 (9)	0.0181 (8)	0.0016 (7)	−0.0051 (7)	0.0070 (7)
N1	0.0279 (13)	0.0181 (11)	0.0208 (12)	0.0025 (10)	0.0016 (10)	0.0021 (9)
C1	0.0222 (14)	0.0143 (12)	0.0224 (14)	0.0011 (10)	0.0016 (11)	−0.0002 (10)
I2	0.01730 (9)	0.02003 (9)	0.01945 (9)	0.00009 (7)	0.00027 (7)	−0.00083 (7)
F2	0.0278 (9)	0.0195 (8)	0.0241 (8)	−0.0002 (6)	0.0041 (7)	0.0074 (6)
N2	0.0216 (12)	0.0219 (12)	0.0209 (12)	−0.0008 (9)	−0.0009 (10)	−0.0006 (10)
C2	0.0225 (14)	0.0240 (14)	0.0178 (13)	0.0069 (11)	0.0000 (11)	−0.0014 (11)
I3	0.01585 (9)	0.02765 (10)	0.03083 (10)	0.00845 (7)	0.00992 (7)	0.00949 (8)
F3	0.0245 (8)	0.0328 (9)	0.0135 (7)	0.0048 (7)	−0.0003 (6)	0.0060 (7)
N3	0.0262 (13)	0.0250 (13)	0.0287 (13)	−0.0008 (10)	−0.0095 (11)	0.0027 (11)
C3	0.0248 (14)	0.0195 (13)	0.0227 (14)	0.0020 (11)	0.0021 (11)	0.0031 (11)
I4	0.02028 (9)	0.01707 (9)	0.02182 (9)	0.00801 (7)	0.00392 (7)	0.00142 (7)
F4	0.0234 (8)	0.0289 (9)	0.0181 (8)	−0.0044 (7)	−0.0027 (7)	−0.0003 (7)
C4	0.0180 (13)	0.0159 (12)	0.0190 (13)	0.0052 (10)	0.0018 (10)	−0.0016 (10)
F5	0.0188 (8)	0.0159 (7)	0.0305 (9)	0.0005 (6)	0.0071 (7)	0.0045 (6)
C5	0.0225 (14)	0.0145 (12)	0.0236 (14)	0.0043 (10)	0.0027 (11)	−0.0004 (11)
F6	0.0127 (8)	0.0240 (9)	0.0551 (12)	0.0025 (6)	0.0081 (8)	0.0028 (8)
C6	0.0207 (13)	0.0178 (13)	0.0222 (14)	0.0080 (10)	0.0021 (11)	−0.0034 (11)
F7	0.0230 (9)	0.0150 (8)	0.0807 (15)	−0.0028 (7)	0.0133 (9)	0.0034 (9)
C7	0.0176 (13)	0.0199 (13)	0.0211 (13)	0.0081 (10)	0.0004 (11)	−0.0032 (11)
F8	0.0280 (9)	0.0184 (8)	0.0603 (12)	0.0122 (7)	0.0135 (9)	0.0039 (8)
C8	0.0162 (13)	0.0177 (13)	0.0238 (14)	0.0012 (10)	0.0009 (11)	−0.0037 (11)
C9	0.0195 (13)	0.0187 (13)	0.0222 (14)	0.0026 (10)	0.0034 (11)	0.0019 (11)
C10	0.0182 (13)	0.0196 (13)	0.0225 (14)	0.0064 (10)	−0.0023 (11)	−0.0016 (11)
C11	0.0206 (14)	0.0186 (13)	0.0270 (15)	−0.0023 (11)	−0.0020 (12)	0.0013 (11)
C12	0.0274 (15)	0.0175 (13)	0.0259 (15)	0.0032 (11)	0.0023 (12)	0.0031 (11)
C13	0.057 (2)	0.041 (2)	0.0320 (18)	−0.0005 (17)	−0.0209 (17)	0.0090 (16)
C14	0.0239 (16)	0.0400 (19)	0.0421 (19)	0.0014 (14)	−0.0120 (14)	−0.0023 (15)
C15	0.0206 (13)	0.0180 (13)	0.0188 (13)	0.0020 (10)	0.0052 (11)	0.0017 (10)
C16	0.0189 (13)	0.0228 (13)	0.0127 (12)	0.0072 (11)	0.0004 (10)	0.0006 (10)
C17	0.0147 (12)	0.0193 (13)	0.0167 (13)	0.0014 (10)	0.0017 (10)	−0.0020 (10)
C18	0.0191 (13)	0.0188 (13)	0.0181 (13)	0.0026 (10)	0.0064 (10)	0.0026 (10)
C19	0.0173 (13)	0.0235 (14)	0.0125 (12)	0.0051 (10)	0.0026 (10)	0.0021 (10)
C20	0.0166 (13)	0.0218 (13)	0.0158 (13)	0.0001 (10)	0.0017 (10)	−0.0018 (10)
C21	0.0126 (12)	0.0230 (13)	0.0168 (13)	0.0052 (10)	0.0041 (10)	0.0036 (10)
C22	0.0166 (12)	0.0167 (12)	0.0147 (12)	0.0022 (10)	0.0020 (10)	0.0017 (10)
C23	0.0166 (12)	0.0166 (12)	0.0184 (13)	0.0061 (10)	0.0034 (10)	0.0003 (10)
C24	0.0123 (12)	0.0212 (13)	0.0264 (14)	0.0025 (10)	0.0038 (11)	0.0014 (11)
C25	0.0205 (14)	0.0151 (13)	0.0370 (17)	0.0007 (11)	0.0053 (12)	0.0018 (12)
C26	0.0233 (14)	0.0149 (13)	0.0256 (14)	0.0068 (11)	0.0052 (11)	0.0031 (11)

### Geometric parameters (Å, º)

**Table:**

I1—C15	2.099 (3)	F8—C26	1.336 (3)
F1—C16	1.348 (3)	C8—C9	1.382 (4)
N1—C2	1.330 (3)	C8—H8	0.9500
N1—C1	1.331 (3)	C9—C10	1.405 (4)
C1—C4	1.390 (4)	C9—H9	0.9500
C1—H1	0.9500	C10—C11	1.408 (4)
I2—C17	2.099 (2)	C11—C12	1.375 (4)
F2—C18	1.347 (3)	C11—H11	0.9500
N2—C2	1.328 (3)	C12—H12	0.9500
N2—C3	1.335 (4)	C13—H13A	0.9800
C2—H2	0.9500	C13—H13B	0.9800
I3—C21	2.074 (2)	C13—H13C	0.9800
F3—C19	1.350 (3)	C14—H14A	0.9800
N3—C10	1.382 (3)	C14—H14B	0.9800
N3—C13	1.451 (4)	C14—H14C	0.9800
N3—C14	1.456 (4)	C15—C20	1.382 (4)
C3—C4	1.392 (4)	C15—C16	1.386 (4)
C3—H3	0.9500	C16—C17	1.383 (4)
I4—C23	2.086 (2)	C17—C18	1.382 (4)
F4—C20	1.343 (3)	C18—C19	1.371 (4)
C4—C5	1.432 (4)	C19—C20	1.383 (4)
F5—C22	1.344 (3)	C21—C26	1.387 (4)
C5—C6	1.196 (4)	C21—C22	1.389 (4)
F6—C24	1.336 (3)	C22—C23	1.378 (4)
C6—C7	1.428 (4)	C23—C24	1.394 (4)
F7—C25	1.341 (3)	C24—C25	1.376 (4)
C7—C8	1.391 (4)	C25—C26	1.377 (4)
C7—C12	1.401 (4)		
			
C2—N1—C1	116.2 (2)	N3—C14—H14A	109.5
N1—C1—C4	122.7 (2)	N3—C14—H14B	109.5
N1—C1—H1	118.7	H14A—C14—H14B	109.5
C4—C1—H1	118.7	N3—C14—H14C	109.5
C2—N2—C3	116.5 (2)	H14A—C14—H14C	109.5
N2—C2—N1	126.5 (3)	H14B—C14—H14C	109.5
N2—C2—H2	116.7	C20—C15—C16	117.2 (2)
N1—C2—H2	116.7	C20—C15—I1	120.76 (19)
C10—N3—C13	118.6 (2)	C16—C15—I1	122.0 (2)
C10—N3—C14	118.7 (3)	F1—C16—C17	118.4 (2)
C13—N3—C14	116.1 (3)	F1—C16—C15	118.2 (2)
N2—C3—C4	122.2 (3)	C17—C16—C15	123.4 (2)
N2—C3—H3	118.9	C18—C17—C16	116.9 (2)
C4—C3—H3	118.9	C18—C17—I2	121.17 (19)
C1—C4—C3	115.8 (2)	C16—C17—I2	121.86 (19)
C1—C4—C5	121.1 (2)	F2—C18—C19	118.1 (2)
C3—C4—C5	123.1 (2)	F2—C18—C17	120.1 (2)
C6—C5—C4	176.0 (3)	C19—C18—C17	121.8 (2)
C5—C6—C7	179.2 (3)	F3—C19—C18	120.7 (2)
C8—C7—C12	117.9 (2)	F3—C19—C20	119.9 (2)
C8—C7—C6	120.9 (2)	C18—C19—C20	119.4 (2)
C12—C7—C6	121.2 (3)	F4—C20—C15	120.5 (2)
C9—C8—C7	121.6 (2)	F4—C20—C19	118.3 (2)
C9—C8—H8	119.2	C15—C20—C19	121.2 (2)
C7—C8—H8	119.2	C26—C21—C22	117.7 (2)
C8—C9—C10	120.7 (3)	C26—C21—I3	120.75 (19)
C8—C9—H9	119.6	C22—C21—I3	121.58 (19)
C10—C9—H9	119.6	F5—C22—C23	118.5 (2)
N3—C10—C9	121.2 (3)	F5—C22—C21	118.6 (2)
N3—C10—C11	121.4 (2)	C23—C22—C21	122.9 (2)
C9—C10—C11	117.4 (2)	C22—C23—C24	117.4 (2)
C12—C11—C10	121.5 (2)	C22—C23—I4	121.67 (19)
C12—C11—H11	119.3	C24—C23—I4	120.92 (19)
C10—C11—H11	119.3	F6—C24—C25	118.3 (2)
C11—C12—C7	120.9 (3)	F6—C24—C23	120.6 (2)
C11—C12—H12	119.6	C25—C24—C23	121.2 (2)
C7—C12—H12	119.6	F7—C25—C24	120.3 (2)
N3—C13—H13A	109.5	F7—C25—C26	119.8 (2)
N3—C13—H13B	109.5	C24—C25—C26	119.8 (2)
H13A—C13—H13B	109.5	F8—C26—C25	118.5 (2)
N3—C13—H13C	109.5	F8—C26—C21	120.5 (2)
H13A—C13—H13C	109.5	C25—C26—C21	121.0 (2)
H13B—C13—H13C	109.5		
